# Vascular dementia: novel insights through multiscale mechanistic modeling

**DOI:** 10.1038/s41392-025-02433-2

**Published:** 2025-10-23

**Authors:** Qiying Ye, Seongjoon Won, Dongming Cai

**Affiliations:** 1 N. Bud Grossman Center for Memory Research and Care, University of Minnesota, Minneapolis, MN USA; 2 Department of Neurology, University of Minnesota Medical School, Minneapolis, MN USA; 3 Geriatric Research Education & Clinical Center (GRECC), Minneapolis VA Health Care System, Minneapolis, MN USA

**Keywords:** Neurological disorders, Diseases of the nervous system

In a recent study published in *Cell*^1^ that combines cell-type-specific transcriptomic profiles generated from a novel vascular dementia (VaD) mouse model with a human VaD single-nuclei RNA-sequencing (sn-RNA-seq) dataset, dysregulated intercellular signaling targets of white matter injury were identified and further validated in vivo. This study opens new directions for future development of biomarkers and/or therapeutics for VaD.

The research team at the University of California at Los Angeles first generated a mouse model that replicates the white matter (WM) ischemia pathology of human VaD.^1^ They demonstrated that intracranial injections of a vasoconstrictor, L-NIO in C57BL/6J mice induced focal ischemia within the subcortical WM regions, disrupting the prefrontal cortex-hippocampal circuit without eliciting widespread damage to the motor cortex. Longitudinal studies over 2 months post-injection indicated behavioral changes such as cognitive and motor deficits, as well as pathological processes such as capillary dilation, oligodendrocyte progenitor cell (OPC) proliferation, myelin loss and ventricular enlargement reminiscent of human VaD-associated hallmarks. They then employed translating ribosome affinity purification (TRAP) approaches using Rpl22-HA (HA-tagged ribosome unit) to isolate ribosome-bound mRNAs from different brain cell types, including endothelial cells (ECs: Tier2-Cre:Rpl22-HA), pericytes/fibroblasts (Tbx18-Cre:Rpl22-HA), oligodendrocyte progenitor cells (OPCs: Ng2-Cre:Rpl22-HA) and astrocytes (GfaABC1D:Rp122-HA). Cell-type-specific transcriptomic analysis revealed diminished expression of canonical markers in WM, defining a transcriptomic profile distinct from cortical tissue.

Furthermore, Tian et al. combined cell-type-specific transcriptomic profiles of the VaD mouse model with a human VaD single-nuclei RNA-sequencing (sn-RNA-seq) dataset^2^ to examine how VaD-related ischemic injury disrupts ligand-receptor (L-R) signaling within the neurovascular niche. They built the largest L–R interaction database to date by merging three existing sources.^3–5^ and applied a six-tier filtering strategy to prioritize candidate pathways. This pipeline ranked ligand–receptor pairs based on ligand identity, cross-species conservation, cell-type specificity, receptor co-regulation, and known neurological relevance. Two top candidates were identified and functionally validated by genetic and/or pharmacological perturbation approaches in vivo: the Serpine2–Lrp1 and the CD39-A3AR signaling pathways. It was found that the expression of Serpine2 was up-regulated in pericytes and microglia of VaD mouse brains, and astrocytes of human VaD brains, binding to Lrp1 on OPCs whose expression was elevated as well, with a resultant inhibition of OPC maturation. Knocking down Serpine2 expression (Serpine2^+/−^) promoted OPC differentiation and remyelination, and improved memory function in the VaD mouse model. In contrary, the CD39–A3AR signaling axis was down-regulated in microglia and endothelial cells in WM lesions of VaD mouse brains, and the CD39 levels were reduced in human VaD samples as well. Over-expression of CD39 via AAV-based gene delivery prior to the VaD induction promoted tissue repair in VaD mice. Pharmacological activation of A3AR using Piclidenoson, a drug being tested in clinical trials for psoriasis treatment, enhanced myelination and restored memory and motor function and rescued pathological changes associated with VaD in vivo, even when administered 5 days after VaD induction (a delayed treatment strategy mimicking clinical scenarios). Together, these studies identified dysregulated intercellular interactomes implicating two signaling pathways as potential therapeutic targets of VaD.

There are several important scientific insights provided by this study (Fig. 1). First, a novel VaD mouse model was generated and characterized as summarized in the paper (Supplemental Fig. S1J by Tian et al.^1^) that recapitulates several key clinical, pathological and cellular features of human VaD conditions, offering a valuable platform for testing future VaD therapeutics (Fig. 1a). However, it should be noted that behavioral assessments of this VaD mouse model were conducted within 2 months post-induction, whereas human VaD progresses over several years and is frequently complicated by Alzheimer’s disease (AD) co-pathology. Moreover, animal studies were carried out in male mice only, which could limit the translatability of observations from this VaD mouse model in this study.

**Fig. 1:**
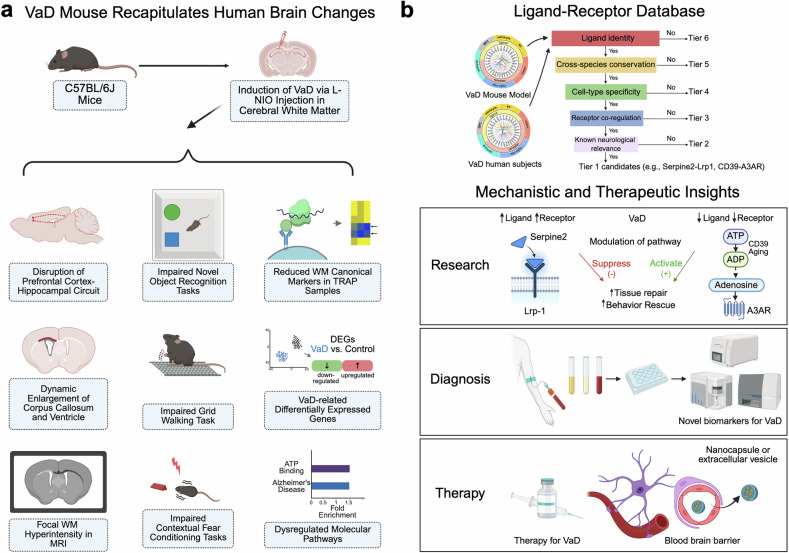
A summary of research studies and findings. **a** A novel VaD mouse model recapitulates key neuropathological, behavior and molecular features observed in human VaD patients. **b** Multiscale data mining identified key signaling changes in mouse and human VaD conditions, which provide mechanistic insights with translational implications for VaD, including future directions for mechanistic investigation, diagnostic and therapeutic development. Created in BioRender. Ye, Q. (2025) https://BioRender.com/xo4vx6v

Secondly, this study introduces a tiered screening approach (Supplementary Fig. S5A by Tian et al.^1^) to dissect high-throughput data and gain mechanistic insights into white matter diseases linking brain cell-type-specific changes to functional outcomes with translational and clinical implications (Fig. 1b). VaD currently lacks reliable molecular biomarkers and thereby identified signaling changes in Serpine2–Lrp1 and CD39-A3AR axis could be explored with future biomarker development. While authors used some post-mortem human brain sample (VaD core and adjacent tissue versus controls) from the NIH brain bank to validate expression changes in these key regulators in infarcted white matter regions of VaD cases, a more comprehensive analysis of these regulators and associated downstream pathway involved in VaD disease mechanisms would be helpful to establish any causal relationship between dysregulated pathways and the initiation of vascular pathological cascades.

Thirdly, the studies with the A3AR-based therapeutics are intriguing as Piclidenoson, an A3AR-specific agonist which has been tested in ongoing clinical trials for psoriasis. However, it was delivered in the VaD mouse models via osmotic pump with resultant functional rescue. Further studies will be needed to explore the bioavailability and blood–brain barrier (BBB) permeability with this drug candidate. Alternatively, strategies such as CNS delivery via nanoparticles or extracellular vesicles and medicinal chemistry modifications of this drug candidate to improve its BBB penetration can be further explored.

Finally, both signaling pathways highlighted in this study, the Serpine2–LRP1 and the CD39–A3AR axis, have been associated with AD, suggesting potential overlaps between VaD and AD pathogenesis. Future studies are needed to determine if these dysregulated signaling pathways are specific to the VaD or commonly shared among various neurodegenerative processes, which can guide the next steps of precision medicine therapeutics development. In summary, this comprehensive study advances our mechanistic understanding of VaD pathological processes and opens new avenues for developing diagnostic and/or therapeutic strategies for VaD patients.

## References

[1] Tian, M. et al. Deconstructing the intercellular interactome in vascular dementia with focal ischemia for therapeutic applications. *Cell* **188**, 1–18 (2025). 10.1016/j.cell.2025.06.002 PMC12221338 40592323

[2] Mitroi, D. N., Tian, M., Kawaguchi, R., Lowry, W. E. & Carmichael, S. T. Single-nucleus transcriptome analysis reveals disease- and regeneration-associated endothelial cells in white matter vascular dementia. *J. Cell Mol. Med.* **26**, 3183–3195 (2022). 35543222 10.1111/jcmm.17315 PMC9170821

[3] Cabello-Aguilar, S. et al. SingleCellSignalR: inference of intercellular networks from single-cell transcriptomics. *Nucleic Acids Res.* **48**, e55 (2020). 32196115 10.1093/nar/gkaa183 PMC7261168

[4] Efremova, M., Vento-Tormo, M., Teichmann, S. A. & Vento-Tormo, R. CellPhoneDB: inferring cell-cell communication from combined expression of multi-subunit ligand-receptor complexes. *Nat. Protoc.* **15**, 1484–1506 (2020). 32103204 10.1038/s41596-020-0292-x

[5] Shao, X. et al. CellTalkDB: a manually curated database of ligand-receptor interactions in humans and mice. *Brief Bioinform*. **22**, bbaa269 (2021). 10.1093/bib/bbaa269 33147626

